# Cerebrovascular diseases in two patients with entire *NSD1* deletion

**DOI:** 10.1038/s41439-021-00151-z

**Published:** 2021-05-24

**Authors:** Toshiyuki Itai, Satoko Miyatake, Taku Hatano, Nobutaka Hattori, Atsuko Ohno, Yusuke Aoki, Kazuya Itomi, Harushi Mori, Hirotomo Saitsu, Naomichi Matsumoto

**Affiliations:** 1 Department of human genetics, Yokohama City University Graduate School of Medicine, Yokohama, Kanagawa Japan; 2 Clinical Genetics Department, Yokohama City University Hospital, Yokohama, Kanagawa Japan; 3 Department of Neurology, Juntendo University, Bunkyo-Ku, Tokyo, Japan; 4 Department of Pediatric Neurology, Toyota Municipal Child Development Center, Toyota, Aichi Japan; 5 Division of Neurology, Aichi Children’s Health and Medical Center, Obu, Aichi Japan; 6 Department of Radiology, School of Medicine, Jichi Medical University, Shimotsuke, Tochigi Japan; 7 Department of Biochemistry, Hamamatsu University School of Medicine, Hamamatsu, Shizuoka Japan

**Keywords:** Clinical genetics, Stroke

## Abstract

We describe two patients with *NSD1* deletion, who presented with early-onset, or recurrent cerebrovascular diseases (CVDs). A 39-year-old female showed developmental delay and abnormal gait in infancy, and developed slowly-progressive intellectual disability and movement disorders. Brain imaging suggested recurrent parenchymal hemorrhages. A 6-year-old male had tremor as a neonate and brain imaging revealed subdural hematoma and brain contusion. This report suggests possible involvement of CVDs associated with *NSD1* deletion.

Sotos syndrome (MIM 117550) is a congenital disorder with the cardinal features of characteristic facial appearance, overgrowth in childhood, and mild to severe learning disability. Haploinsufficiency of *NSD1* is a major cause of Sotos syndrome^1^. *NSD1* (MIM 606681) at 5q35 encodes Nuclear receptor-binding SET domain protein 1 (NSD1), which interacts with nuclear receptors and acts as both a coactivator and a corepressor^2,3^. *NSD1* abnormalities, including missense variants, frameshift variants, nonsense variants, splice site variants, as well as large submicroscopic deletions involving the entire *NSD1* gene at 5q35.2–q35.3, have been reported with an apparent phenotype–genotype correlation between phenotype severity and single nucleotide variant/short indel or large deletion^4^.

To date, more than 450 Sotos syndrome patients with an *NSD1* abnormality have been reported in Human Gene Mutation Database Professional (as of 17 February 2021). Their clinical features have been well characterized and include the cardinal features, dysmorphic facial appearance, learning disability, and childhood overgrowth, as well as advanced bone age, cardiac anomalies, renal anomalies, and scoliosis. Central nervous system symptoms of macrocephaly, ventriculomegaly, behavioral problems, and seizures have also been reported^4^, although only one patient has been described with cerebrovascular disease (CVD)^5^. Here we report two Sotos syndrome patients arising from entire *NSD1* deletion that have CVDs.

Two Sotos patients with CVDs were recruited for genetic investigation. Clinical information was obtained from medical doctors and medical records. Experimental protocols were approved by the IRB of Yokohama City University Faculty of Medicine. Informed consent was obtained from their guardians.

WES was performed for Patient 1 and her parents, as previously described^6^. Targeted sequencing for *COL4A1/COL4A2* was performed for Patient 2, as previously described^7^.

Exome CNV analysis was performed using the eXome-Hidden Markov Model (XHMM)^8^ for Patient 1, and single nucleotide polymorphism (SNP) microarray analysis using the CytoScan HD Array (Affymetrix, Santa Clara, California) for Patient 2 according to the manufacturer’s protocol.

qPCR of the target region was performed using genomic DNA and a QuantiFast SYBR Green PCR kit (Qiagen, Hilden, Germany) on a Rotor-Gene Q real-time PCR cycler (Qiagen). Relative ratios of genomic DNA copy number between the patient, the parents, and the normal control were calculated using Rotor‐Gene 6000 Series Software 1.7 (Qiagen) and the standard curve method. Duplicate experiments were performed. Primer sequences are available upon request.

Patient 1 was the third child born to healthy nonconsanguineous Japanese parents with no family history (Fig. 1a). There was no perinatal complication. In the neonatal period, feeding difficulty was recognized. At the age of 3 months, developmental delay was found at her regular check-up. She started walking at the age of two years and six months with a supportive device. She attended special support classes at schools. At the age of 17 years, she had tonic-clonic seizures. She had an epileptic stroke once a month, even after starting to take phenytoin. She was able to communicate verbally and had a wide-based gait, but gradually regressed. At the age of 21 years, she could hardly walk because of ataxia, and her ability to speak was reduced. Brain magnetic resonance imaging (MRI) revealed leukoencephalopathy. Alexander disease was suspected, but *GFAP* screening was negative. At the age of 39 years, a brain MRI suggested recurrent episodes of parenchymal hemorrhage (Fig. 1b, c).

**Fig. 1:**
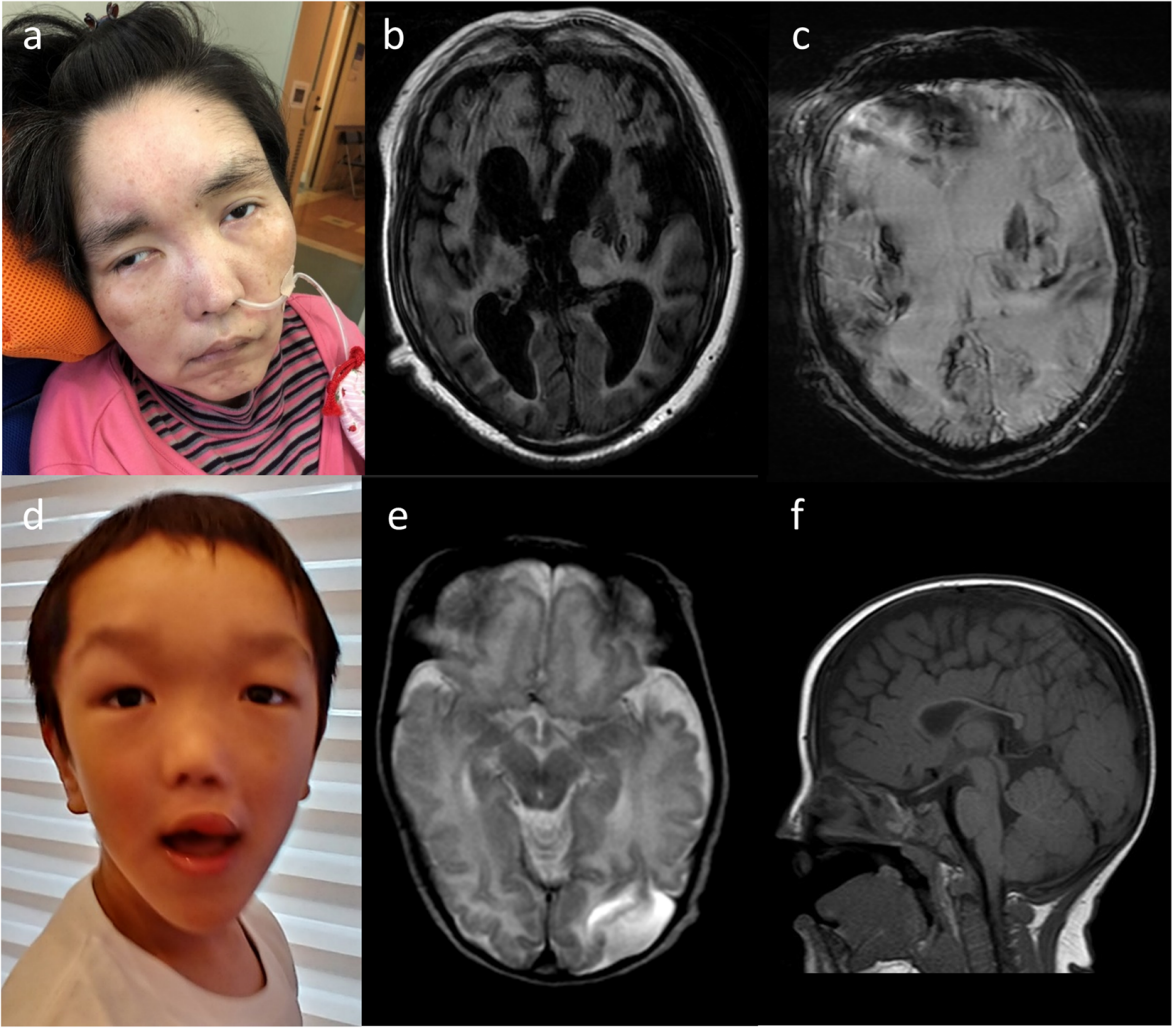
Clinical features of Patients 1 and 2. **a** A facial photograph of Patient 1 at 39 years of age showing sparse eyebrows and a pointed chin. Brain MRI at 39 years of age. **b** Fluid-attenuated inversion recovery (FLAIR) imaging showed bilateral enlarged lateral ventricles and reduced white matter volume with high intensity. **c** T2*-weighted gradient recalled echo imaging showed multiple low-intensity areas in the cerebral cortex and basal ganglia. **d** A facial photograph of Patient 2 at 6 years of age showing macrocephaly and characteristic facial features, including down-slanted palpebral fissures and a pointed chin. **e** Brain T2-weighted MRI at 12 days showed subdural hematoma in the left posterior lobe, parenchymal hemorrhage, partial brain atrophy of the left posterior lobe, and reduced white matter volume. **f** T1-weighted imaging showed thin corpus callosum.

Patient 2, a 6-year-old boy, was the second child born to healthy nonconsanguineous Japanese parents with no family history (Fig. 1d). During pregnancy, he was suspected to have cleft lip and palate by fetal ultrasonography. He was delivered vaginally at 41 weeks and 5 days of gestation with a birth weight of 3970 g (+1.89 SD) and head circumference of 38 cm (+3.37 SD). Apgar scores at 1 and 5 min were 8 and 9, respectively. Cleft lip and palate were confirmed after birth. In addition, he had macrocephaly, characteristic facial features including down-turned palpebral fissures, umbilical hernia, patent foramen ovale, and hydrocele. He was admitted to a neonatal intensive care unit for controlling feeding difficulties associated with cleft lip and palate. In the early neonatal period, he had transient tachypnea, and blood oxygen saturation (SpO_2_) was decreased to ~90%; hence he was treated with 30% oxygen supplementation. He also presented with tremors, but no abnormal electroencephalogram findings were observed. Brain MRI at the age of 12 days revealed subdural hematoma, parenchymal hemorrhage in the occipital lobe area, and thin corpus callosum (Fig. 1e, f). Blood tests for coagulation and protein C activity were within the normal ranges, and there was no abnormal autoantibody toward thrombophilia. G-band karyotyping was normal. His developmental milestones were delayed: head control at 5 months, sitting still at 7 months, independent walking and speaking significant words were both achieved at the age of 2 years 9 months. He gradually showed a pointed chin. He entered a special support class at elementary school.

WES was performed for Patient 1. We detected no pathogenic SNV associated with CVD, but found a 1.6 Mb de novo deletion at 5q35.2–q35.3 (Chr5:175,511,998–177,180,174, GRCh37/hg19) encompassing *NSD1* using XHMM (Fig. 2a, b). qPCR confirmed that the deletion occurred de novo (Fig. 2c). In Patient 2, due to the existence of early-onset parenchymal hemorrhages and seizures, targeted sequencing was performed to screen the genes associated with hereditary cerebral small vessel diseases, including *COL4A1/COL4A2* as previously described^7^, but no pathogenic variant was detected. Then a SNP microarray analysis was performed because of dysmorphic features, and a 1.9 Mb deletion at 5q35.2–q35.3 (Chr5: 175,570,677–177,437,651) involving *NSD1* was detected (Fig. 2d). qPCR confirmed that the deletion occurred de novo (Fig. 2e).

**Fig. 2:**
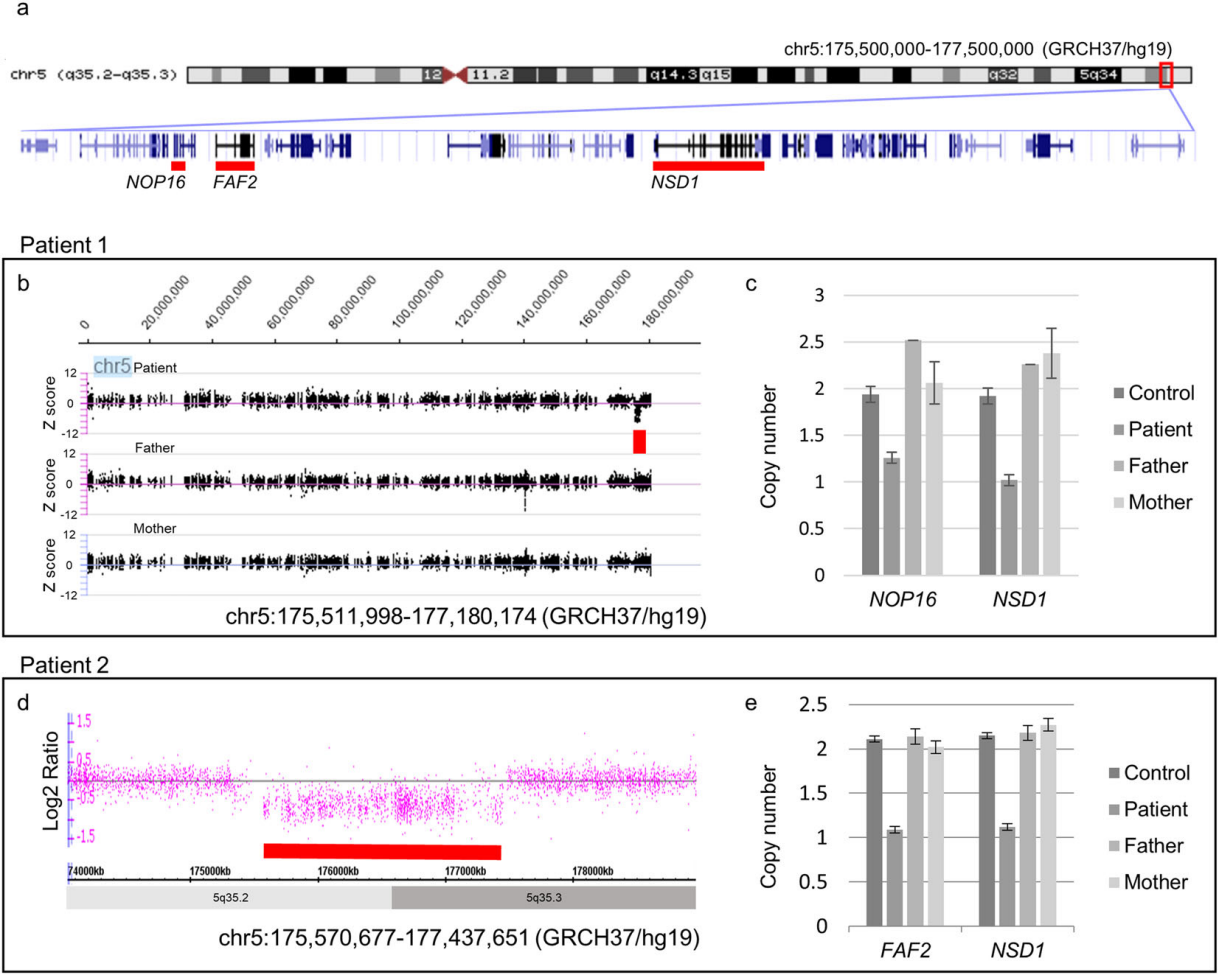
CNV analyses in Patients 1 and 2. **a** A schematic presentation of the 5q35.2–q35.3 genomic region. *NSD1* (at 5q35.3), *NOP16* (at 5q35.2), and *FAF2* (at 5q35.2) are shown with red underbars. CNV analysis in Patient 1. **b** XHMM showed *de novo* deletion at chr5:175511998–177180174. *X*-axis; genomic position, *Y*-axis, Z score. **c** qPCR showed that the deletion occurred de novo. *X*-axis; targeted genes, *Y*-axis; copy number. Bars show the mean ± standard error. CNV analysis in Patient 2. **d** Microarray analysis showed deletion at chr5:175570677–177437651. *X*-axis; genomic position, *Y*-axis; log_2_ ratio of copy number. **e** qPCR showed that the deletion occurred de novo.

We report two patients of Sotos syndrome accompanied by CVDs. To our knowledge, one other patient of Sotos syndrome with CVD has been described who had recurrent chronic subdural hematoma, and harbored a de novo canonical splice site variant in *NSD1* (NM_022455.4:c.5892+1G>T)^5^. Collectively, therefore, three of >450 patients of Sotos syndrome showed CVDs (<0.6%).

The diagnosis of Sotos syndrome is generally made from distinctive facial appearances during infancy to early childhood, followed by genetic testing^9^. In contrast, both of our patients had their genetic diagnoses prior to the clinical diagnoses. Our results suggest that *NSD1* abnormalities can be identified in unexpected situations, particularly in individuals with cerebrovascular diseases in early infantile periods or in adult patients whose Sotos syndrome was unnoticed.

Our two patients, together with the patient reported by Carli et al.^5^, indicate that parenchymal hemorrhage and subdural hematoma may be characteristic features of CVDs associated *NSD1* abnormality. Given that the precise pathogenesis of CVD is unclear, it is indeed difficult to explain the observed difference of disease onset and CVD phenotype. Furthermore, the overlapped deleted regions of patients 1 and 2 (chr5:175,570,677-177,180,174) includes at least following five genes highly intolerant to null variants that have not been associated with human diseases: *FAF2* (MIM 616935), *DBN1* (MIM 126660), and *FAM193B* (MIM 615813) showing ubiquitous expression patterns including the vasculature; *GPRIN1* (MIM 611239) and *UNC5A* (MIM 607869) abundantly expressed in the central nervous system (gnomAD, https://gnomad.broadinstitute.org/; GTExPortal, https://www.gtexportal.org/). Deletion of either of these genes may contribute to CVDs.

The long-term prognosis of Sotos syndrome is not fully understood. Although clinical evaluation of 44 adults with Sotos syndrome suggested that the long-term prognosis of Sotos syndrome is generally good^10^, more patients are needed to conclude the long-term prognosis, especially when relatively rare complications are apparent. In this context, CVDs may be an important prognostic factor because CVDs are generally associated with poor prognosis and impaired quality of life. The current clinical management of Sotos syndrome does not usually include assessment for CVDs^9^. However, brain assessment should be considered if patients have neurovascular symptoms.

In conclusion, we suggest that CVDs are a rare complication of Sotos syndrome. Further patients are needed to confirm this observation.

## Data Availability

All required data are included in the manuscript.

## HGV database

The relevant data from this Data Report are hosted at the Human Genome Variation Database at 10.6084/m9.figshare.hgv.300910.6084/m9.figshare.hgv.3012
